# Sunitinib as Second‐Line Treatment in Advanced Intrahepatic Cholangiocarcinoma: Results From the SUN‐CK GERCOR Phase II Trial

**DOI:** 10.1111/liv.70196

**Published:** 2025-07-09

**Authors:** Louis Gros, Mohamed Bouattour, Clément Dumont, Laëtitia Dahan, David Malka, Annemilaï Tijeras‐Raballand, Armand De Gramont, Maxime Ronot, Chantal Dreyer, Cindy Neuzillet, Philippe Bourget, Alexandra Hadengue, Nelly Roldan, Marie‐Line Garcia‐Larnicol, Benoist Chibaudel, Eric Raymond, Sandrine Faivre

**Affiliations:** ^1^ Department of Medical Oncology Paris Saint‐Joseph Hospital Group Paris France; ^2^ Department of Oncology Centre Hospitalier Universitaire Vaudois (CHUV), Lausanne University Lausanne Switzerland; ^3^ Department of Liver Cancer and Innovative Therapy Unit, Hôpital Beaujon, Assistance Publique‐Hôpitaux de Paris, AP‐HP. Nord, Clichy, France; Université Paris Cité, Centre de Recherche Sur L'Inflammation (CRI), INSERM, U1149, CNRS Paris France; ^4^ Medical Oncology Department, Saint‐Louis Hospital, Assistance Publique‐Hôpitaux de Paris, AP‐HP. Nord, Paris, France; Université Paris Cité, Centre de Recherche Sur L'Inflammation (CRI), INSERM, U1149, CNRS Paris France; ^5^ Service Oncologie Digestive & Hépato‐Gastroentérologie CHU Timone, Assistance Publique‐Hôpitaux de Marseille (APHM) & Aix‐Marseille‐University (AMU) Marseille France; ^6^ Department of Medical Oncology, Institut Mutualiste Montsouris, Paris, France; Unité Dynamique des Cellules Tumorales, INSERM U1279, Université Paris‐Saclay, Gustave Roussy, Villejuif, France; GERCOR Paris France; ^7^ OncoMEGA Lamorlaye France; ^8^ AFR Oncology Boulogne‐Billancourt France; ^9^ Department of Radiology, Hôpital Beaujon, Assistance Publique‐Hôpitaux de Paris, AP‐HP. Nord, Clichy, France; Université Paris Cité, Centre de Recherche Sur L'Inflammation (CRI), INSERM, U1149, CNRS Paris France; ^10^ Institut de Cancérologie et Radiothérapie Bretillien Saint‐Malo France; ^11^ Gastrointestinal Oncology, Medical Oncology Department, Institut Curie, Université Versailles Saint‐Quentin‐Université Paris‐Saclay, Saint‐Cloud, France; Molecular Oncology, PSL Research University, CNRS, UMR 144, Institut Curie Paris France; ^12^ Department of Clinical Pharmacy, Hôpital Necker‐Enfants Malades, AP‐HP Paris France; ^13^ GERCOR‐IRC Paris France; ^14^ Department of Medical Oncology, Hôpital Franco‐Britannique ‐ Fondation Cognacq‐Jay, Cancérologie Paris Ouest Levallois‐Perret France

**Keywords:** angiogenesis inhibitors, biliary tract cancer, Choi criteria, tumour density, VEGF

## Abstract

**Background & Aims:**

Angiogenesis is critical in intrahepatic cholangiocarcinoma (ICC), a highly lethal cancer with limited treatment options. Sunitinib, a multi‐receptor tyrosine kinase inhibitor, has strong antiangiogenic and antitumor effects. We aimed to evaluate the efficacy and tolerability of sunitinib as a second‐line treatment in chemotherapy‐pretreated patients with advanced ICC.

**Methods:**

This open‐label, single‐arm, phase II trial was conducted across five French centres. Eligible patients were aged ≥ 18, had advanced ICC not suitable for curative surgery and had been pre‐treated with gemcitabine and/or platinum. Patients received oral sunitinib at 37.5 mg daily. The primary endpoint was overall survival (OS), with secondary endpoints including progression‐free survival (PFS), objective response rate (ORR), disease control rate (DCR) and safety. Tumour response was evaluated using RECIST v1.1, with exploratory analysis using Choi criteria. VEGF‐A, VEGF‐C and plasma sunitinib levels were measured.

**Results:**

Fifty‐three patients were included. Median OS was 9.6 months (95% CI, 6.1–13.1), median PFS was 3.7 months (95% CI, 3.1–6.4), the ORR was 14% and the DCR was 84%. Grade 3–4 cytopenias occurred in 25% of patients, hypertension in 21% and fatigue in 19%. Higher baseline levels of VEGF‐A and VEGF‐C correlated with longer OS. Patients with dose reductions maintained adequate drug exposure. Choi criteria, applied to 24 patients, predicted response duration better than RECIST v1.1. Baseline tumour density, assessed in 14 patients, showed a potential association with treatment responses.

**Conclusions:**

Sunitinib demonstrated efficacy and manageable toxicity as a second‐line treatment in patients with advanced ICC previously treated with chemotherapy, suggesting it may represent a viable therapeutic option for this population.

**Trial Registration:**

ClinicalTrials.gov NCT01718327

**Summary**

- Intrahepatic cholangiocarcinoma is an increasingly diagnosed cancer with a high mortality rate.
- We conducted a study to test the tolerance and effectiveness of sunitinib in patients whose disease progressed after initial chemotherapy treatment.
- Our results suggest that sunitinib is a promising treatment option that could potentially extend the lives of some patients.

**Abbreviations**
AE: adverse event
BOR: best overall response
cKIT: stem‐cell factor receptor
CR: complete response
CT: computed tomography
CTCAE: Common Terminology Criteria for Adverse Events
DCR: disease control rate
ECOG PS: Eastern Cooperative Oncology Group Performance Status
FGF2: fibroblast growth factor 2
HGF: hepatocyte growth factor
MRI: magnetic resonance imaging
OPN: osteopontin
ORR: objective response rate
OS: overall survival
PD: progressive disease
PFS: progression‐free survival
PR: partial response
RECIST: Response Evaluation Criteria In Solid Tumours
SD: stable disease
SDF1: stromal cell‐derived factor 1
SPARC: secreted protein, acidic, cysteine‐rich
ULN: upper limit of normal
VEGF: vascular endothelial growth factor

## 1 Introduction

Biliary tract cancer (BTC) is a rare and heterogeneous group of malignancies arising from the epithelial cells lining the biliary tract. It includes intrahepatic, perihilar and distal cholangiocarcinoma (CC), as well as gallbladder cancer. Intrahepatic cholangiocarcinoma (ICC) is the second most common primary hepatic malignancy, with its incidence and mortality rates having increased dramatically over the past decades [1]. ICC is often diagnosed at advanced stages when curative surgery is no longer feasible [2]. For patients with advanced disease, the median overall survival (OS) is less than 12 months [3, 4]. Despite significant progress made in understanding the molecular pathogenesis of ICC, its management remains challenging.

Platinum‐based chemotherapy, specifically the combination of gemcitabine and cisplatin, has been the first‐line standard of care for more than a decade based on the results from the ABC‐02 and BT22 phase III trials [5, 6]. Recently, immunotherapy combined with chemotherapy has emerged as a new standard of care for ICC treatment, showing improvement of OS in two phase III trials [7, 8]. The ABC‐06 study demonstrated improved OS with second‐line 5‐fluorouracil‐leucovorin‐oxaliplatin (FOLFOX) compared to supportive care alone [9]. However, these treatments enable only a minority of patients to achieve long‐term disease control, emphasising the need for more effective treatments.

Molecular profiling identifies actionable mutations in nearly half of patients with ICC, leading to the investigation of various targeted therapies. As a result, drugs such as pemigatinib, futibatinib (FGFR2 inhibitors) and ivosidenib (IDH1 inhibitor) have been approved for patients with previously treated, unresectable locally advanced or metastatic ICC [10, 11, 12, 13]. However, the outcomes of molecular‐targeted therapies remain moderate and are limited to specific patient populations. Approximately 40% of patients exhibit molecular aberrations that align with FDA‐approved therapeutic options, yet effective strategies are still needed for a significant proportion of patients with advanced ICC who lack actionable mutations [14, 15].

One of the therapeutic strategies involves inhibiting angiogenesis, as angiogenesis and lymphangiogenesis are critical regulators in the progression of cholangiocarcinoma, particularly ICC [16]. High microvessel density has been associated with lower curative resection rates and local recurrence in patients with ICC [16, 17]. Cholangiocyte growth and dissemination are facilitated by an extensive blood vessel network fostered by factors secreted by the cholangiocytes themselves [18]. While vascular endothelial growth factor (VEGF)‐A is the main regulator of angiogenesis [19], VEGF‐C and VEGF‐D play key roles in promoting lymphangiogenesis [20].

Sunitinib, an orally available multiple receptor tyrosine kinase inhibitor (TKI), has demonstrated potent antiangiogenic and antitumor activity, prolonging survival in several angiogenesis‐dependent cancers [21, 22]. In a previous study, we reported favourable tolerability and sustained disease control in selected patients with ICC treated with sunitinib [23]. Based on these findings, we designed the SUN‐CK phase II trial to evaluate the efficacy and safety of sunitinib as a second‐line treatment for patients with advanced ICC.

In a translational approach, we investigated angiogenesis‐related biomarkers as potential predictors of response to sunitinib and assessed tumour size and density using the Choi criteria [24], which may offer greater accuracy than RECIST v1.1 (Response Evaluation Criteria in Solid Tumors) in hypervascularised tumours [25, 26, 27, 28].

## 2 Patients and Methods

### 2.1 Study Design

The SUN‐CK study was a phase II, open‐label, single‐arm, multicenter trial (NCT01718327) conducted in five French centres. The protocol (in accordance with the CONSORT statement) was approved by the institutional review board or the local independent ethics committee and was conducted in accordance with the International Conference on Harmonization Good Clinical Practice guidelines of the Declaration of Helsinki and applicable local regulatory requirements and local laws. All participants provided written consent.

### 2.2 Patient Eligibility

This trial included patients aged 18 or older with histologically or cytologically proven, locally advanced (unresectable) or metastatic ICC whose disease had progressed after gemcitabine and/or platinum‐based chemotherapy [25]. Additional main inclusion criteria were measurable disease according to RECIST v1.1, an Eastern Cooperative Oncology Group performance status (ECOG PS) of 0 or 1, a life expectancy of at least 3 months and adequate organ function, including adequate liver function (alanine transaminase, aspartate transaminase, alkaline phosphatase levels < 5 times the upper limit of normal values (ULN), bilirubin < 1.5 ULN). Key exclusion criteria included known central nervous system metastases, uncontrolled hypertension, abnormal cardiac function and any of the following events within the previous 12 months: myocardial infarction, severe/unstable angina, symptomatic congestive heart failure, cerebrovascular accident or transient ischaemic attack, or pulmonary embolism. Full inclusion and exclusion criteria can be found in the complete protocol.

### 2.3 Study Treatment

Eligible patients received oral sunitinib at a dose of 37.5 mg once daily until disease progression, unacceptable toxicity, withdrawal of consent or premature discontinuation of treatment. Sunitinib was withheld for any grade 3–4 toxicity until it resolved to grade ≤ 2. For grade 4 or recurrent grade 3 toxicity, the dose was reduced to 25 mg once daily. Patients who experienced a delay of > 3 consecutive weeks due to toxicity discontinued from sunitinib treatment.

### 2.4 Endpoints and Assessments

The primary endpoint was OS, defined as the time from the first dose of sunitinib administration to death from any cause. Secondary endpoints included progression‐free survival (PFS), defined as the time from the first dose of sunitinib to proven radiological disease progression or death from any cause; best overall response (BOR), defined as the best response recorded from the start of treatment until disease progression or recurrence; objective response rate (ORR), defined as the percentage of all patients who experienced a complete response (CR) or partial response (PR) and disease control rate (DCR), defined as the proportion of patient who had CR, PR or stable disease (SD). The duration of SD was measured from the start of treatment until the criteria for disease progression were met.

Prior to treatment initiation, patients underwent radiological baseline (computed tomography [CT] or magnetic resonance imaging [MRI]) assessments within 28 days. Physical examination and laboratory testing were performed every 2 weeks during the first month of treatment and subsequently every 4 weeks until the end of progression.

Adverse events were graded according to the National Cancer Institute Common Terminology Criteria for Adverse Events (NCI‐CTCAE) v4.0 [29].

Tumour response was evaluated by CT or MRI according to RECIST v1.1 at baseline and every 8 weeks until the end of treatment. In addition, a post hoc exploratory analysis was conducted using imaging data to compare response assessments between RECIST v1.1 and the Choi criteria [24], including comparisons of PFS according to radiological responses and an investigation of lesion density as a predictive factor for response. Post‐treatment, patients underwent clinical evaluation every 2 months.

Serum levels of angiogenesis‐related biomarkers (VEGF‐A, VEGF‐C, osteopontin, soluble KIT [sKIT], stromal‐derived factor 1 [SDF1], serum protein acidic and rich in cysteine [SPARC], fibroblast growth factor 2 [FGF2] and hepatocyte growth factor [HGF]) were assessed using enzyme‐linked immunosorbent assays (ELISA) at baseline and monthly until treatment discontinuation.

Plasma concentrations of sunitinib and its active metabolite, N‐desethyl sunitinib (SU12662), were assessed prior to treatment and monthly until treatment discontinuation using validated high‐performance liquid chromatography, with detection limits of 10–250 ng/mL for sunitinib and 15–250 ng/mL for SU12662 [30]. Total drug concentrations were calculated as the sum of sunitinib and SU12662 concentrations.

### 2.5 Statistical Analysis

The primary objective was to test the null hypothesis that the hazard rate, assumed to be constant across all study intervals, was identical between historical results and the studied protocol. The study design included an accrual period of 18 months and a follow‐up period of 6 months. The null hypothesis (H0) assumed a median OS of 3 months, consistent with historical data [31, 32], while the alternative hypothesis (H1) proposes an expected median OS of 6.3 months. Using one‐arm non‐parametric survival with a two‐sided alpha of 5% and a statistical power of 91%, it was required to include 51 patients. The computation assumed an attrition rate of 0.05 per month, which was separated from the censoring of subjects that occurred when the study ended.

OS and PFS data were summarised using the Kaplan–Meier estimator, and medians were estimated accordingly. Response rates were presented as percentages.

As no pre‐established cut‐off values were available for angiogenesis‐related biomarkers, patients were stratified into two groups based on the median baseline serum levels of each marker. The log‐rank test was used to compare OS between patients with high and low baseline serum levels of angiogenesis‐related markers.

In our exploratory analysis, we used descriptive statistics to compare response assessments between RECIST v1.1 and the Choi criteria, the latter of which defines response as a decrease in the largest diameter of at least 10 percent or a reduction in in tumour density of at least 15 percent on CT [24, 25]. The Fisher's exact test was used to compare frequencies, while the Kruskal‐Wallis rank sum test was applied to analyse differences in PFS based on response assessments. Spearman's correlation analysis was conducted to assess the association between the mean baseline density of target lesions and response assessments, as well as variations in target lesion density.

All statistical analyses were performed with SAS (SAS Institute, Cary, NC).

## 3 Results

### 3.1 Study Population

**FIGURE 1:**
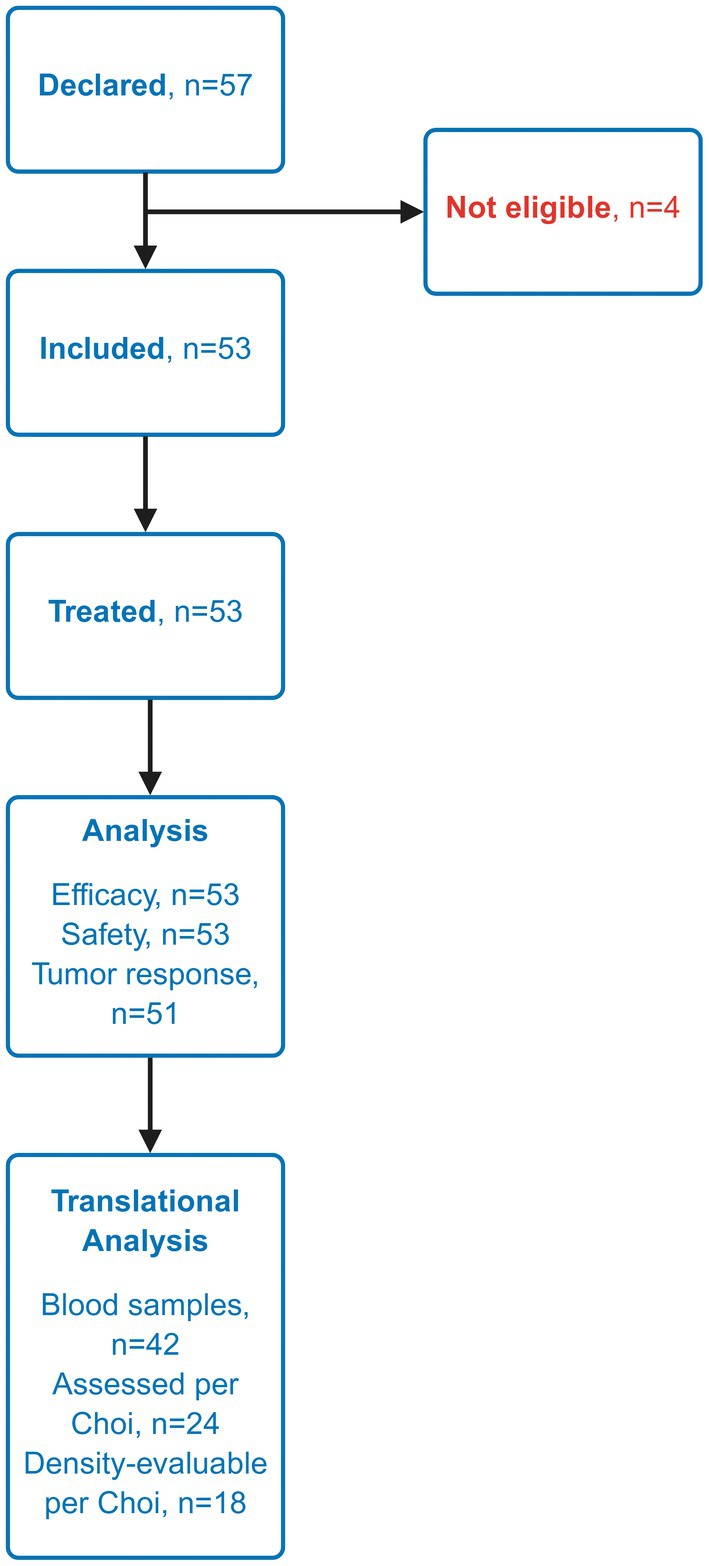
Flowchart of the study.

Out of 57 patients screened, 53 were enrolled and included in the study (Figure 1). All 53 patients received at least one dose of sunitinib and were included in both the OS and safety analyses. Two patients were excluded due to non‐cancer death before the first response assessment, leaving 51 patients evaluable for tumour response. Table 1 summarises the patient baseline demographic and disease characteristics.

**TABLE 1:** Baseline demographics and clinical characteristics.

	Patient population *n* = 53	%
Age, years		
Median, range	61 (27–81)	
< 70	49	92
≥ 70	4	8
Sex		
Female	25	47
Male	28	53
ECOG PS		
0	33	62
1	20	38
Previous surgery		
No	27	51
Yes	26	49
Surgical margin status		
R0	12	23
R1 or R2	14	26
Previous adjuvant systemic therapy		
Yes	12	23
No	41	77
Metastatic sites		
Liver	13	25
Lung	6	11
Peritoneum	3	6
Lymph node	3	6
Bone	1	2
Other	2	4
Median size of target lesions at inclusion, cm (95% CI)	6.8 (1.3–20.5)	
Previous chemotherapy		
Adjuvant treatment	9	17
First line for advanced treatment	44	83

Abbreviation: ECOG PS, Eastern Cooperative Oncology Group Performance Status.

### 3.2 Efficacy

**FIGURE 2:**
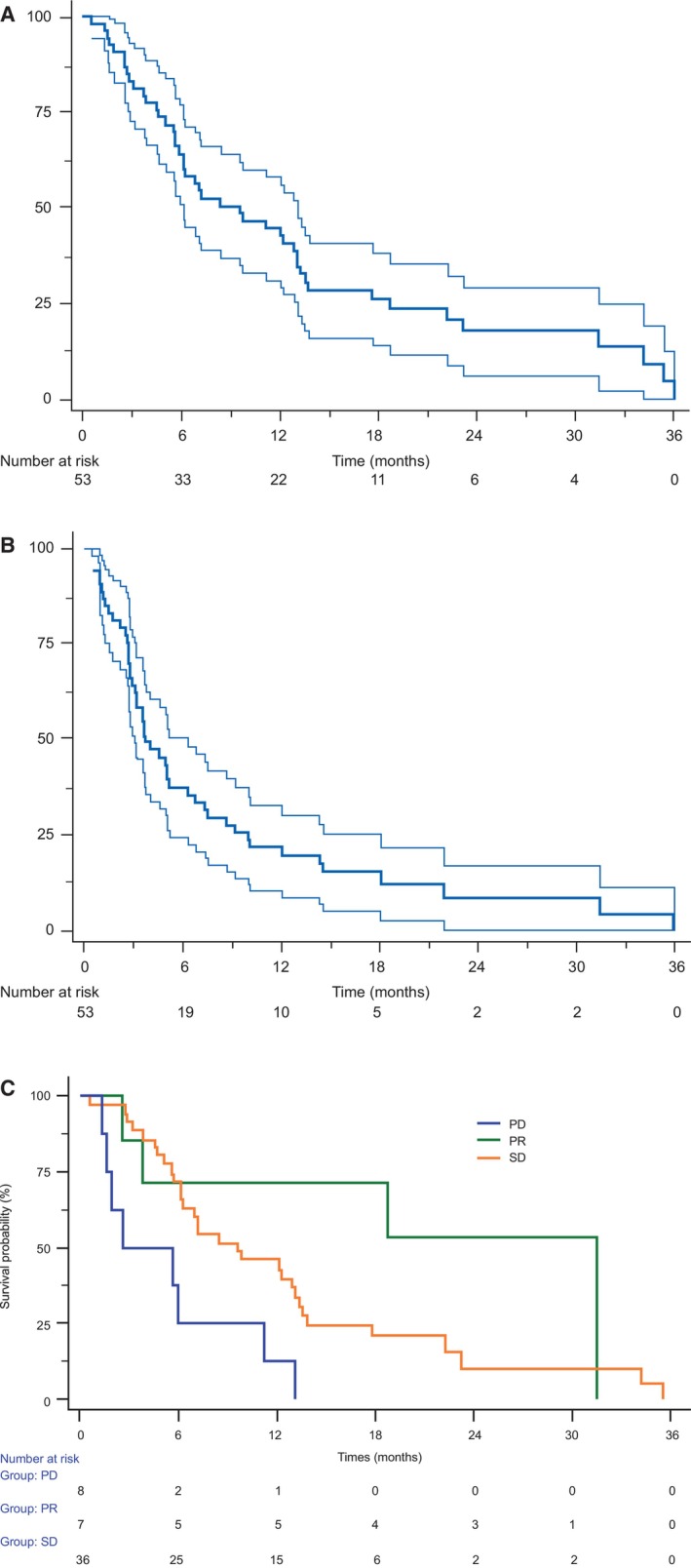
(A) Overall survival, (B) Progression‐free survival and (C) Overall survival based on best overall response.

The median OS was 9.6 months (95% CI, 6.1–13.1), which met the per‐protocol statistical boundaries for anti‐tumour activity (Figure 2A). The median PFS was 3.7 months (95% CI, 3.1–6.4) (Figure 2B). Among the 51 patients evaluated for tumour response, seven patients experienced an objective response (ORR, 14%) and 36 had their disease stabilised (DCR, 84%; Table 2). Figure 2C shows the OS of patients according to the best overall response after sunitinib treatment. The median OS of patients who experienced PR was 31.5 months (95% CI, 3.8‐NR). Patients with SD had a median OS of 9.6 months (95% CI, 6.2–13.1), which was significantly superior (*p* < 0.0024) to that of patients with progressive disease (PD), who had a median OS of only 2.6 months (95% CI, 1.6–11.2).

**TABLE 2:** Best tumour response.

Response	Patient population (%)
ORR	7 (14)
CR	0 (0)
PR	7 (14)
SD	36 (71)
PD	8 (16)
DCR	43 (84)

Abbreviations: CR, complete response; DCR, disease control rate; ORR, objective response rate; PD, progressive disease; PR, partial response; SD, stable disease.

### 3.3 Safety

After a mean treatment duration of 5.7 months, safety findings were consistent with the known safety profile of sunitinib (Table 3). The most frequently reported grade 1–2 adverse events were fatigue (68%), diarrhea (58%), abdominal pain (41%) and hand‐foot syndrome (40%). Grade 3–4 (asymptomatic) neutropenia and thrombocytopenia were observed in about one‐quarter of patients. Grade 3–4 hypertension was observed in 11 (21%) patients, while 10 (19%) patients experienced grade 3–4 fatigue. Most toxicities were manageable with either symptomatic treatment or dose reduction to 25 mg, which was required for 34 (45.3%) patients, most commonly for thrombocytopenia (38%) and asthenia (30%). One patient experienced a fatal gastric perforation during the study, which was considered treatment‐related by both the investigator and the sponsor.

**TABLE 3:** Adverse events.

Worst grade	1 *n* (%)	2 *n* (%)	3 *n* (%)	4 *n* (%)	5 *n* (%)	All *n* (%)
Haematologic						
Thrombocytopenia^a^	28 (53)	8 (15)	11 (21)	2 (4)	0 (0)	49 (92)
Anaemia	21 (40)	14 (26)	5 (9)	0 (0)	0 (0)	40 (75)
Lymphopenia	20 (38)	8 (15)	8 (15)	1 (2)	0 (0)	37 (70)
Neutropenia*	7 (13)	15 (28)	12 (23)	2 (4)	0 (0)	36 (68)
Non‐haematologic						
Fatigue	19 (36)	17 (32)	10 (19)	0 (0)	0 (0)	46 (87)
Weight loss	10 (19)	2 (4)	0 (0)	0 (0)	0 (0)	12 (23)
Nausea	13 (24)	4 (7)	1 (2)	0 (0)	0 (0)	18 (34)
Vomiting	13 (24)	4 (7)	2 (4)	0 (0)	0 (0)	19 (36)
Diarrhoea	21 (40)	10 (19)	1 (2)	0 (0)	0 (0)	32 (60)
Constipation	7 (13)	3 (6)	0 (0)	0 (0)	0 (0)	10 (19)
Stomatitis	10 (19)	10 (19)	2 (4)	0 (0)	0 (0)	22 (42)
Dysgeusia	12 (23)	5 (9)	0 (0)	0 (0)	0 (0)	17 (32)
Hand‐food syndrome	15 (28)	6 (11)	2 (4)	0 (0)	0 (0)	23 (43)
Hypertension	4 (7)	4 (7)	11 (21)	0 (0)	0 (0)	19 (36)
Epistaxis	6 (11)	0 (0)	0 (0)	0 (0)	0 (0)	6 (11)
Abdominal pain	10 (19)	12 (23)	1 (2)	0 (0)	0 (0)	23 (43)
Gastric perforation	0 (0)	0 (0)	0 (0)	0 (0)	1 (2)	1 (2)

^a^ No febrile neutropenia or bleeding was observed.

### 3.4 Plasma Biomarkers

Among 42 (79%) patients with available biomarker data, high baseline levels of VEGF‐A and VEGF‐C were associated with longer OS (median 13.7 vs. 8.4 months; VEGF‐A: 95% CI, 9.6–23.2; *p* = 0.052; VEGF‐C: 95% CI, 9.6–34.2; *p* = 0.024) (Figure S1). In contrast, plasma concentrations of osteopontin, sKIT, SDF1, SPARC, FGF2 and HGF were not associated with OS (Table S1).

### 3.5 Pharmacokinetics

Sunitinib dose adjustment from 37.5 to 25 mg in cycles 1–3 resulted in a decrease in the mean plasma concentration of the total drug from 152 ng/mL (range, 102–241) to 91 ng/mL (range, 39–138), while still maintaining a relevant therapeutic exposure (> 50 ng/mL) [33].

### 3.6 RECIST v1.1 and the Choi Criteria

Among 24 out of 53 (45.3%) patients for whom tumour response was assessed by both RECIST v1.1 and Choi criteria, three patients (12.5%) achieved a PR according to RECIST v1.1, compared to 10 patients (42%) according to the Choi criteria. When incorporating Choi's density criteria, which requires a decrease in tumour density of 15% or more (measured at the portal phase after contrast injection), an additional six patients (16 in total, 67%) showed PR. Overall, RECIST v1.1 classified one patient out of 24 as having PD, 20 as SD and three as PR. In contrast, the Choi criteria classified two patients as PD, six as SD and 16 as PR. The Choi classification was significantly correlated with response duration, unlike RECIST v1.1 (Table 4). However, six out of 24 (25%) patients had CT scans that were non‐evaluable for density due to technical limitations. Of these, five exhibited an infiltrative pattern of ICC and one had target lesions confined to the lungs.

**TABLE 4:** Comparison of response duration by Choi Criteria and RECIST v1.1 (*n* = 24).

	Response duration (months)		*P‐value*
	SD	PR	
Choi (SD)	3.85 [3.27; 4.62]	9.45 [6.60; 12.0]	0.021*
RECIST v1.1 (SD)	7.60 [4.40; 10.1]	13.4 [8.68; 18.2]	0.69

* As the number of subjects compared was small, a non‐parametric test was carried out (Mann–Whitney test).

**FIGURE 3:**
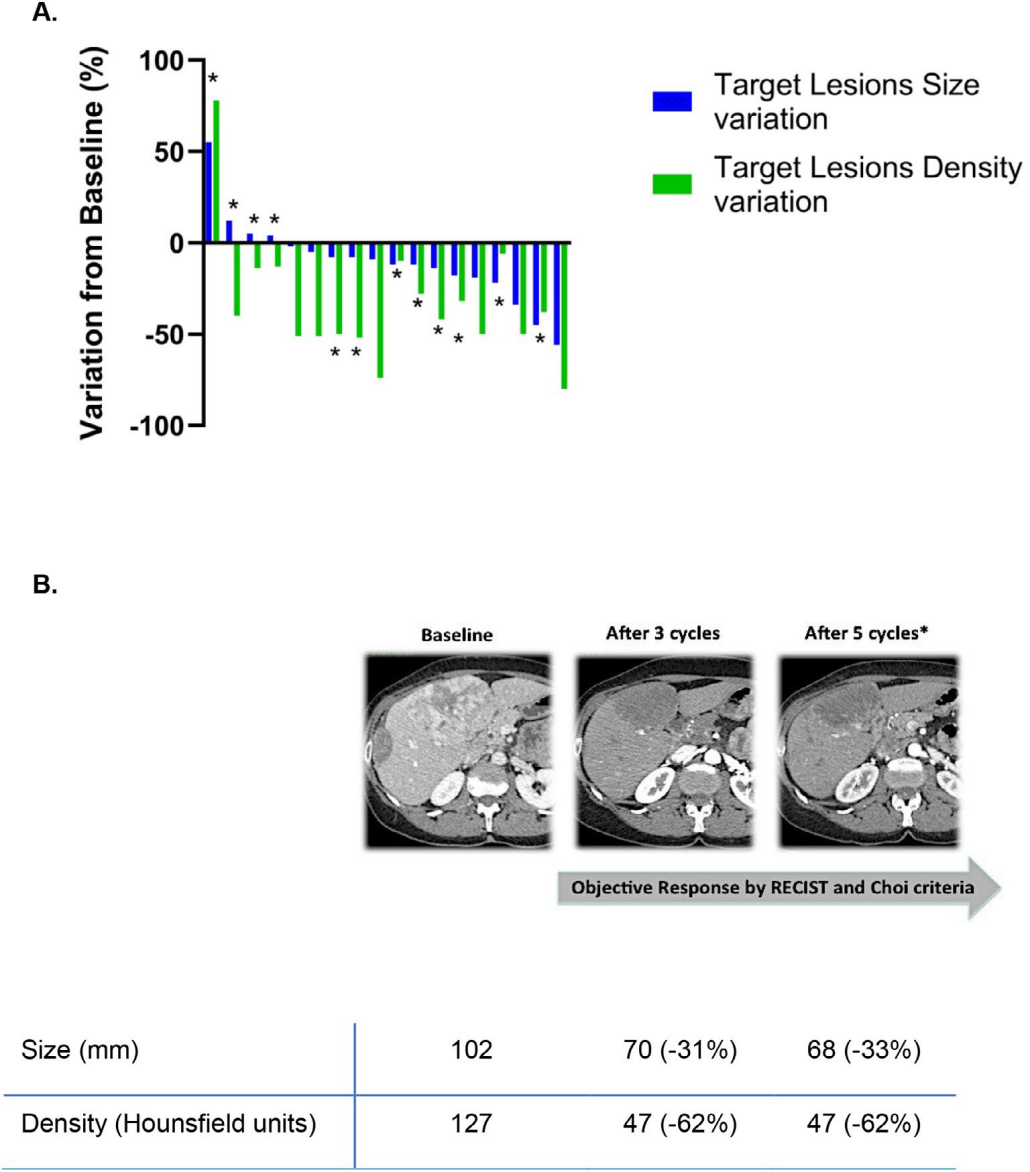
(A) Comparison of radiological assessment according to Choi Criteria versus RECIST v1.1. The asterisks highlight 12 patients who were identified as having partial response based on Choi criteria but were classified as having stable disease as per RECIST v1.1. (B) An example of radiological evaluation demonstrating changes in tumour size and density in one patient after sunitinib treatment.

Figure 3A highlights the differences between RECIST v1.1 and the Choi criteria among the 18 patients evaluable by both size and density. Notably, 12 patients marked with an asterisk (*) were classified as SD by RECIST v1.1 but were reclassified as PR according to Choi criteria. Figure 3B illustrates the effect of sunitinib in a responding patient, demonstrating both tumour shrinkage and a reduction in density.

In the 18 patients with measurable tumour density, the mean baseline density of target lesions correlated with response according to Choi criteria (Table 5). Baseline density showed modest correlations with density variation (*R* ^2^ = 0.29) and overall response to sunitinib (*R* ^2^ = 0.13), highlighting a potential trend. (Figure S2A,B).

**TABLE 5:** Comparison of mean baseline lesion density by Choi Criteria (*n* = 18).

Best overall response according to Choi	Baseline density pattern Hounsfield units (HU)	*P‐value*
PR (SD)	84 (32)	0.048*
SD (SD)	43 (2.1)	
PD (SD)	28 (−)	

* Kruskal‐Walli's rank sum test.

## 4 Discussion

The results of this phase II trial suggest that sunitinib is active in the second‐line treatment of patients with ICC after platinum‐ or gemcitabine‐based chemotherapy. The median OS of 9.6 months (95% CI, 6.1–13.1) and the median PFS of 3.7 months (95% CI, 3.1–6.4) compare well with the median OS of 6.5 months and the median PFS of 2.6 months for second‐line therapies (including chemotherapy and targeted therapy) found in a meta‐analysis of 23 prospective phase II trials and nine retrospective studies, which included a total of 1391 patients with refractory BTC [34]. The ORR of 14% further validated the anti‐tumour activity of sunitinib, superior for example to the ORR of 5% observed with FOLFOX in the second‐line ABC‐06 trial [9]. However, patients with ICC have been shown to have a slightly better prognosis than patients with other types of BTC [14]. These encouraging results suggest that VEGF‐targeted therapies may improve outcomes for patients with advanced ICC.

In a previous phase II study conducted in Asia, sunitinib (37.5 mg daily) was assessed as second‐line therapy for metastatic BTC in 56 patients, 62.5% of whom had ICC [35]. The observed median time to progression of 1.7 months, median OS of 4.8 months and ORR of 8.9% are numerically lower than those observed in our study. The authors attributed their findings to a high incidence of grade 3 or 4 adverse events, which limited treatment exposure. This observation aligns with previous studies on sunitinib‐induced toxicity in Asian patients [36, 37]. In contrast, our study primarily enrolled Caucasian patients, which may explain the better safety outcomes we observed. We hypothesise that the improved efficacy observed in our study is due to enhanced sunitinib exposure and a focus on patients with ICC rather than all BTC.

Overall, the activity of several other multi‐targeted TKIs—for example, lenvatinib, apatinib, regorafenib and sorafenib—in patients with advanced CC who had previously received systemic chemotherapy has been modest [38, 39, 40, 41]. However, these trials have involved diverse patient populations and tumour types, making direct comparisons challenging. We attribute our better results to the exclusive enrollment of ICC patients, the good tolerance of sunitinib and its potent anti‐tumour effects. Although these findings may not be directly translated to all cases of ICC, a meta‐analysis has shown that sunitinib has shown superior survival benefits over sorafenib in the first‐line treatment of metastatic renal cell carcinoma [42]. These observations should be interpreted with caution, and further comprehensive studies are warranted to provide further insights into this matter.

Sunitinib demonstrated a generally manageable side effect profile in our study, consistent with previous reports. Most sunitinib‐related adverse events were manageable through symptomatic treatment, temporary interruption or dose reduction to 25 mg/day. However, one patient died experiencing a gastrointestinal perforation related to sunitinib, a rare event that has been reported in less than 1% of patients receiving sunitinib therapy. The underlying mechanism for this complication remains unclear [43].

Sunitinib administered at a fixed dose showed a substantial inter‐patient variation in exposure and had a low therapeutic index (50–100 ng/mL), while also demonstrating a positive correlation between its dose and effectiveness [30, 44]. We assessed the total trough levels of sunitinib, as levels below 50 ng/mL have been associated with reduced efficacy [30]. For patients requiring a dose reduction, monitoring revealed that the drug levels remained in the appropriate range. This highlights the potential for monitoring sunitinib pharmacokinetics to reduce doses and toxicity without compromising therapeutic effectiveness, aligning with findings from previous clinical trial [30, 44].

We found that patients with higher‐than‐median baseline levels of VEGF‐A and VEGF‐C had significantly longer OS, suggesting that these markers may serve as predictive indicators for benefiting from VEGFR‐targeted therapy in this otherwise poor‐prognosis population. Due to the study's design, it remains unclear whether the correlation between elevated plasma levels of VEGF‐A and VEGF‐C and extended survival with sunitinib reflects an inherently more responsive subgroup of patients. The relationship between the production of VEGF protein isoforms in tumours and its concentration in the circulation is also unclear, and no significant relationship has been observed between circulating VEGF levels and pathologic features [45]. A meta‐analysis of seven studies on VEGF expression and prognosis in ICC showed that high VEGF expression was associated with poor OS [46]. The measurement of VEGF in the circulation as a prognostic marker needs further evaluation, as the cell‐associated isoform (VEGF189), rather than the soluble isoforms (VEGF121 and VEGF165), seems to play an important role in tumour progression [47]. Confirmatory data from other studies with VEGFR‐TKIs would be valuable. Except for VEGF, plasma biomarkers showed no clear association with overall survival; their prognostic value warrants further evaluation in adjusted or multiparametric models.

Anti‐angiogenic therapies induce tumour necrosis and often result in disease stabilisation rather than regression. In an exploratory analysis, we found that Choi criteria were valuable in identifying radiological responses, complementing RECIST v1.1 in detecting responders to sunitinib in advanced ICC patients, in line with previous findings in hepatocellular carcinoma, ICC and pancreatic neuroendocrine tumours treated with sunitinib [26, 48, 49, 50]. Our analysis showed that the Choi criteria were more effective than RECIST v1.1 in categorising response assessments and appeared to predict PFS more accurately. Furthermore, the baseline density of tumour lesions appeared to be potentially associated with response to sunitinib, particularly regarding the duration and magnitude of response, although this observation remains exploratory and requires further validation. These findings align with existing literature, where contrast‐enhanced CT density has been shown to predict the response to sunitinib therapy in other tumours [51]. Although prospective data are needed, our study supports the use of Choi criteria and tumour density measurements to better predict sunitinib response.

Our study has limitations, including a small sample size. This study was conducted prior to the approval of checkpoint inhibitors and targeted therapies. As a result, patients received first‐line chemotherapy alone, which does not fully align with current treatment guidelines recommending the addition of immunotherapy to chemotherapy. However, the combination of VEGFR‐targeted TKI with anti‐PD‐(L)1 immunotherapy is nowadays standard of care in several malignancies, with no significant concerns about synergistic toxicity, alleviating concerns about safety of VEGFR‐TKIs in immunotherapy‐pretreated patients. Further studies are needed to determine the optimal sequencing of these therapies and their potential role in combination with TKIs. Moreover, given the limited size of the cohort, the analysis of VEGF levels as a prognostic factor should be interpreted with caution. Although larger studies may be difficult due to the rarity of the condition, validation in independent datasets remains essential.

In conclusion, the results of our phase II study suggest the activity and tolerability of sunitinib as a second‐line therapy for gemcitabine‐ and/or platinum‐pretreated advanced ICC. Higher levels of VEGF‐A and VEGF‐C were associated with longer OS, highlighting their potential as predictive biomarkers. The Choi criteria outperformed RECIST v1.1 in predicting survival, with baseline density emerging as a predictive factor for response. Further studies are required to confirm these findings, determine optimal treatment strategies and explore the relationships between sunitinib response, drug exposure, plasma angiogenic growth factors and tumour density on CT scans in advanced ICC. Molecular profiling, with a focus on angiogenesis signatures, may play a role in future patient selection.

## Ethics Statement

The protocol (in accordance with the CONSORT statement) was approved by the institutional review board or the local independent ethics committee and was conducted in accordance with the International Conference on Harmonization Good Clinical Practice guidelines of the Declaration of Helsinki and applicable local regulatory requirements and local laws. All participants provided written consent.

## Conflicts of Interest

Mohamed Bouattour reports consulting fees from AstraZeneca, Bayer, Bristol‐Myers Squibb, Eisai, Ipsen, MSD, Roche and Sirtex Medical; speakers bureau from Bayer, Eisai and Roche and support for travel/attending meetings from AstraZeneca, Bayer and Sirtex Medical. Clément Dumont reports occasional engagements for Astellas, BMS, Janssen, MSD and Pfizer. David Malka has received consulting fees/honoraria from AbbVie, Amgen, AstraZeneca, Bayer, BMS, Foundation Medicine, Incyte, Leo Pharma, Merck Serono, MSD, Pierre Fabre Oncologie, Roche, Sanofi, Servier, Taiho and Viatris and travel expenses from Amgen, Bayer, BMS, Merck Serono, MSD, Pierre Fabre Oncologie, Roche, Sanofi, Servier and Viatris. Annemilaï Tijeras‐Raballand Employment: AAREC Filia Research. Travel, Accommodations, Expenses: Merck KGaA. Armand De Gramont Employment: AAREC Filia Research. Consulting or Advisory Role: PharmaEngine Travel, Accommodations, Expenses: Celgene. Maxime Ronot Honoraria from Alexion Pharmaceuticals, Canon‐Toshiba, GE Healthcare, Guerbet, Ipsen, Servier, Sirtex, Terumo; support for meetings from Baxter. Cindy Neuzillet has received consulting fees/honoraria from Amgen, Astellas, AstraZeneca, AAA, Baxter, Bristol‐Myers Squibb, Boehringer Ingelheim, Fresenius Kabi, Incyte Biosciences, Merck, MSD, Mundipharma, Nestlé Health Science, Novartis, Nutricia, OSE Immunotherapeutics, Pierre Fabre, Roche, Sanofi, Servier, Viatris; research funding (to institution) from AstraZeneca, Bristol‐Myers Squibb, Fresenius Kabi, Nutricia, OSE Immunotherapeutics, Roche, Servier, Viatris. Benoist Chibaudel has had a consulting or advisory role with Bayer, Lilly, Roche and Sanofi and has received travel, accommodations and expenses from Amgen, Lilly, Merck, Roche and Sanofi. Eric Raymond reports serving as a consultant for SCOR, Genoscience Pharma, StromaCare and Onward therapeutic and is a shareholder for SCOR, Genoscience Pharma, StromaCare and Axoltis. Sandrine Faivre is a consultant for and has received clinical trials grants from, Bristol‐Myers Squibb, Bayer Pharma, MSD, Novartis and Pfizer.

## Supporting information


Figure S1.

Table S1.

## Data Availability

The data that support the findings of this study are available from the corresponding author upon reasonable request.
