# Supplementary material for: Sunitinib as Second‐Line Treatment in Advanced Intrahepatic Cholangiocarcinoma: Results From the SUN‐CK GERCOR Phase II Trial
Source: Liver Int. 2025 Jul 9;45(8):e70196. doi: 10.1111/liv.70196 (PMC12239060; doi:10.1111/liv.70196)
Supplement: Supplementary file 1 — Figure S1. [file LIV-45-0-s001.docx]

Supplementary Figure 1. Overall survival according to VEGF-A (A) and VEGF-C (B) levels (low versus high) at baseline (n = 42).

**A**


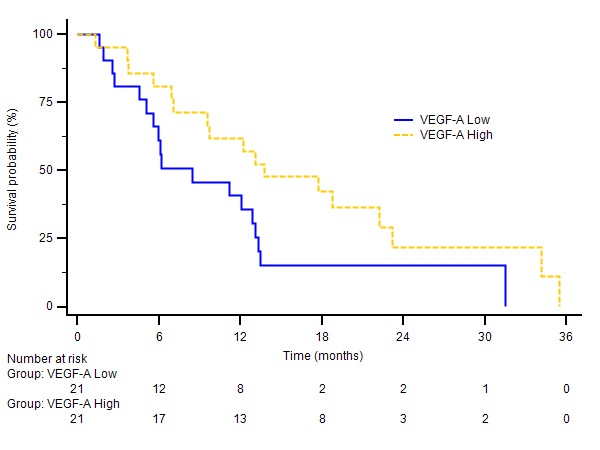


**B**


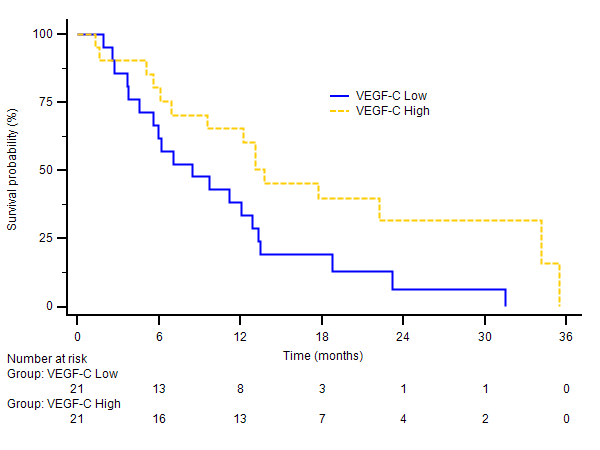


**Supplementary Figure 2A.** Target lesions density variation (best response) according to baseline in patients treated with sunitinib.


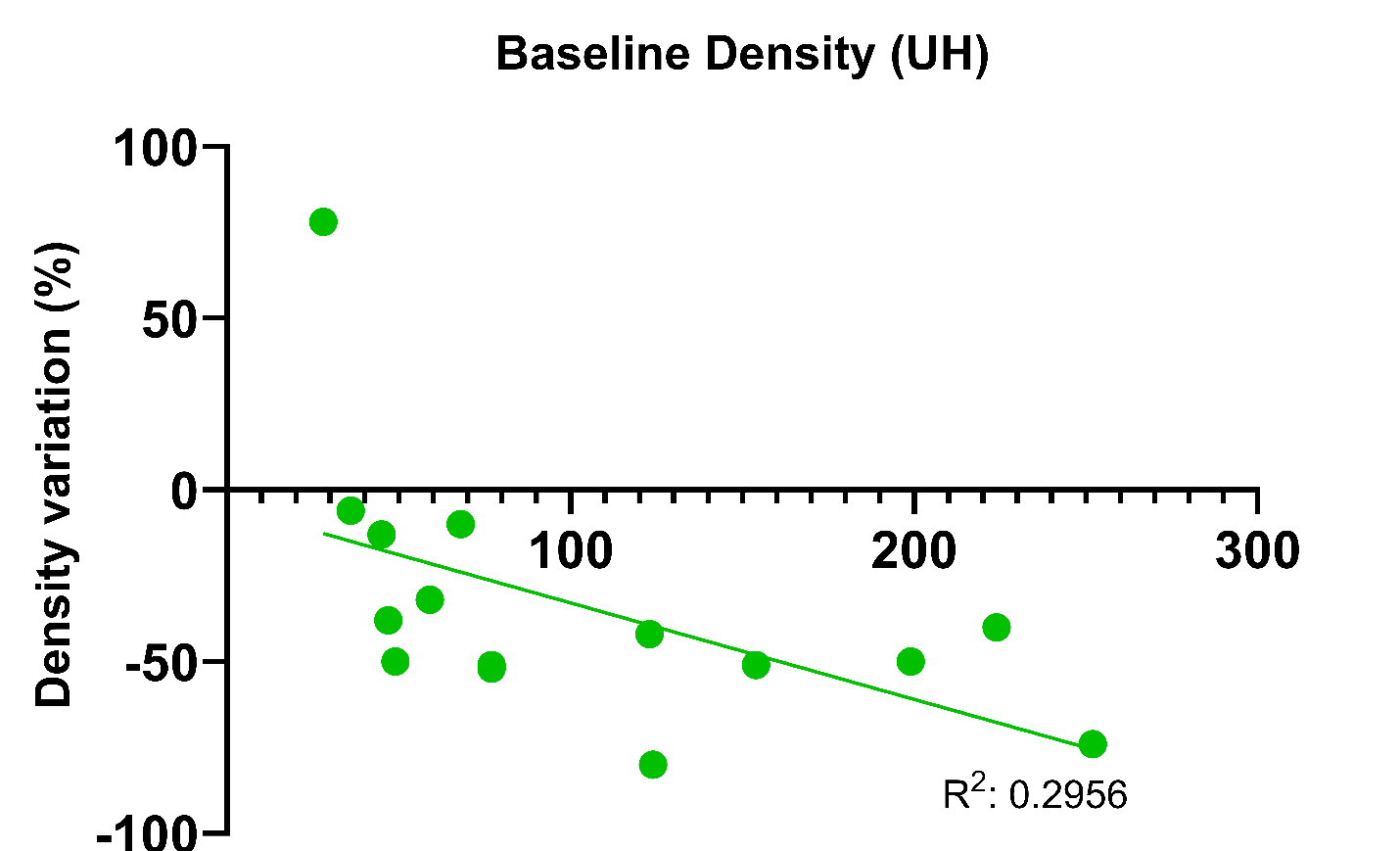


Each point represents one patient. Density variation corresponds to the maximal change observed during treatment. The coefficient of determination (R² = 0.2956) indicates a modest correlation between baseline density and response.

**Supplementary Figure 2B.** Association of Baseline Density With Response to Sunitinib.

**
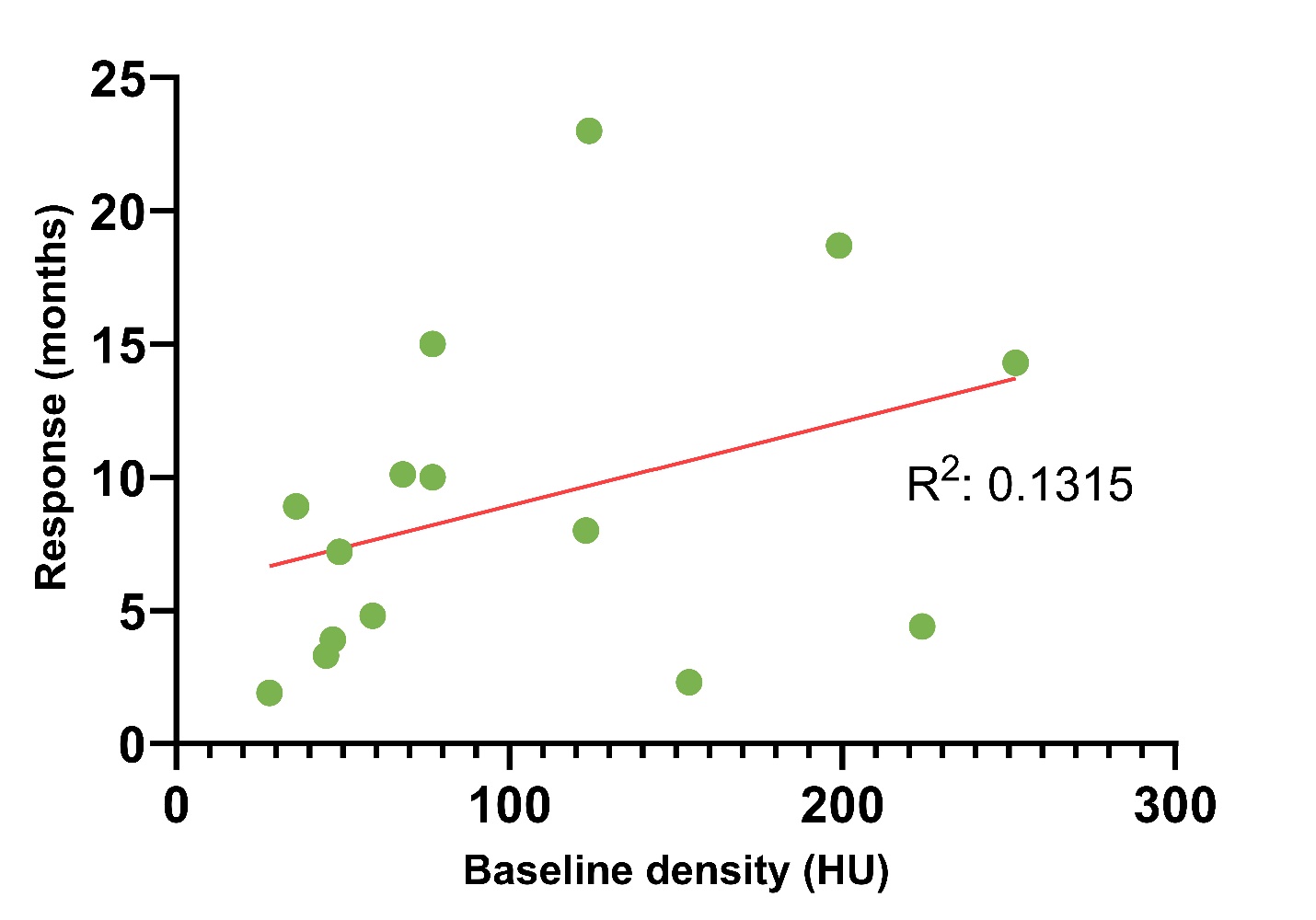
**

Linear regression analysis of baseline density and response to sunitinib, showing a modest association (R² = 0.1315). Each point represents one patient, highlighting a potential trend between initial lesion characteristics and treatment outcome.
