# Supplementary material for: Sunitinib as Second‐Line Treatment in Advanced Intrahepatic Cholangiocarcinoma: Results From the SUN‐CK GERCOR Phase II Trial
Source: Liver Int. 2025 Jul 9;45(8):e70196. doi: 10.1111/liv.70196 (PMC12239060; doi:10.1111/liv.70196)
Supplement: Supplementary file 2 — Table S1. [file LIV-45-0-s002.docx]

**APPENDICES/ SUPPLEMENTARY MATERIAL**

Table 1: Prognostic value of biomarkers for overall survival.

|  |  | Median | 95% CI | HR | 95% CI | *p* |
| --- | --- | --- | --- | --- | --- | --- |
| VEGF-A | Low | 8.4 | 5.6-13.1 | 1 |  |  |
|  | High | 13.7 | 9.6-23.2 | 0.53 | 0.27-1.06 | .052 |
| VEGF-C | Low | 8.4 | 5.6-12.9 | 1 |  |  |
|  | High | 13.7 | 9.6-34.2 | 0.48 | 0.24-0.95 | .024 |
| SDF1 | Low | 9.7 | 6.1-13.1 | 1 |  |  |
|  | High | 13.7 | 6.9-23.2 | 0.622 | 0.318-1.215 | .142 |
| HGF | Low | 11.2 | 5.6-18.7 | 1 |  |  |
|  | High | 12.9 | 7.1-17.7 | 0.89 | 0.45-1.75 | .727 |
| c-KIT | Low | 9.7 | 5.9-17.7 | 1 |  |  |
|  | High | 12.2 | 7.1-13.5 | 1.1 | 0.57-2.14 | .763 |
| FGF2 | Low | 12.1 | 3.8-17.7 | 1 |  |  |
|  | High | 34.2 | 12.2-35.5 | 0.47 | 0.11-2.03 | .213 |
| SPARC | Low | 12.2 | 12.1-35.5 | 1 |  |  |
|  | High | 5 | 1.3-34.2 | 2.02 | 0.53-7.75 | .261 |
| OPN | Low | 11.2 | 5.9-23.2 | 1 |  |  |
|  | High | 13.1 | 3.7-18.7 | 1.1 | 0.54-2.26 | .779 |

Abbreviations: VEGF, vascular endothelial growth factor; cKIT, stem-cell factor receptor; HGF, hepatocyte growth factor; SDF1, stromal cell-derived factor 1; FGF2, fibroblast growth factor 2; SPARC, secreted protein, acidic, cysteine-rich; OPN, osteopontin
